# Ascending Tonic Clonic Seizure Syndrome after Percutaneous Vertebroplasty

**DOI:** 10.1155/2015/870810

**Published:** 2015-04-21

**Authors:** Guido Zarattini, Adam Farrier, Federico Sibona

**Affiliations:** ^1^Orthopaedic Clinic, University of Brescia, Piazzale Spedali Civili 1, 25123 Brescia, Italy; ^2^North Tees University Hospital, Stockton TS19, UK

## Abstract

*Background Context*. Cement leakage is not a rare complication of vertebroplasty, but ascending tonic clonic seizure syndrome is exceptionally rare. We herein report the first case to our knowledge of this complication related to vertebroplasty. *Purpose*. We herein report the first case of ascending tonic clonic seizure syndrome following epidural cement leakage after percutaneous vertebroplasty in a patient with multiple osteoporotic compression fractures. *Study Design*. Case report. *Methods*. A 64-year-old woman with T8, T10, L2, and L4 osteoporotic compression fractures underwent percutaneous vertebroplasty using polymethylmethacrylate. 40 minutes after the procedure the patient started suffering back and leg pain, having repetitive myoclonic jerks lasting 15 seconds of the lower extremities, spasm of the back, dyspnea, sinus tachycardia, hypoxemia, and metabolic acidosis. *Results*. The patient recovered completely due to a combination of early effective resuscitation and considered definitive management. *Conclusions*. Percutaneous vertebroplasty with polymethylmethacrylate is relatively safe but has few dangerous complications, which should be prevented by a meticulous technique and excellent image quality.

## 1. Introduction

Percutaneous vertebroplasty (PV) is a minimally invasive technique that is used to manage osteoporotic vertebral body compression fractures and vertebral metastatic lesions. Complications of vertebroplasty are low, ranging from 2%, when treating osteoporotic compression fractures, to 10% in cases related to malignant tumors [1]. The main complication after this procedure is cement leakage that is reported from 11% to 76% of cases in the literature [2]. Fortunately, it is well tolerated in the large majority of patients. However, cement extravasation is also the main source of clinical complications [3]. Here we present a woman who had ascending tonic clonic seizure syndrome (ATCS) following cement leakage after PV. ATCS is a rare syndrome characterized by painful tonic clonic spasms, lasting approximately 15–30 seconds, accompanied by sinus tachycardia, tachypnea, and metabolic acidosis [4]. ATCS has previously been described in the literature after accidental injection of ionic contrast media for myelography [4]. This is the first reported case, to our knowledge, of ATCS following PV.

## 2. Case Presentation

**Figure 1:**
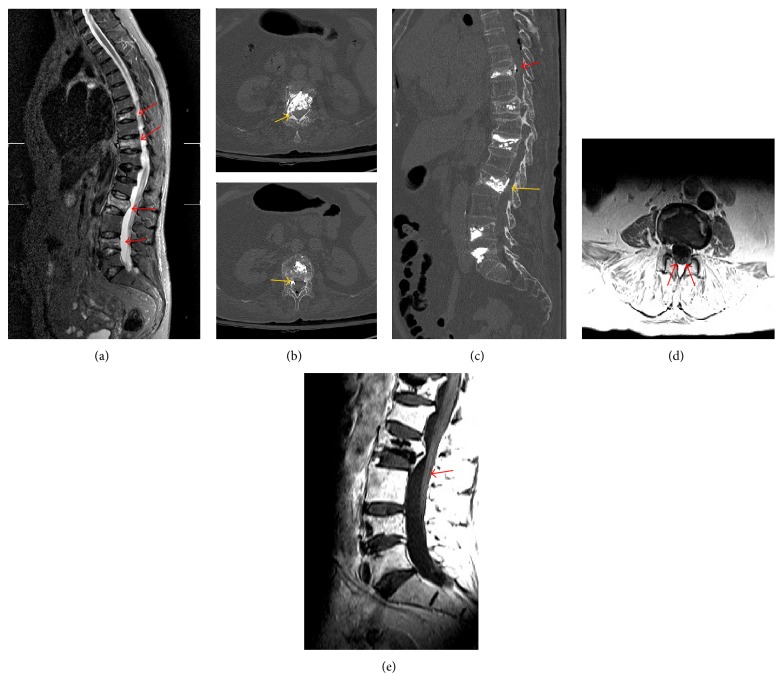
(a) Preinterventional STIR MRI showing acute D8, D10, L2, and L4 vertebral body fracture (red arrows). (b) Postoperative axial CT scan showing epidural cement leakage into the right epidural space at the L2 vertebral level (yellow arrows). (c) Postoperative sagittal CT scan showing epidural cement leakage into the right epidural space at the L2 vertebral level (yellow arrow). Minimal leakage is seen at T8 level (red arrow). (d) Postoperative axial T1, (e) sagittal T1 contrast MRI, showing enhancement of nerve roots along cauda equina (red arrows), signalling meningeal irritation.

A 64-year-old woman (65 kg, 160 cm) with recent T8, T10, L2, and L4 osteoporotic compression fractures confirmed by hyperintense signal in STIR sequence at MRI (Figure 1(a)) was treated with PV at T8, T10, L2, and L4 level because of refractory pain after 4 weeks of conservative treatment. The procedure was done under general anesthesia, using polymethylmethacrylate (PMMA; volume injected 3 cm^3^; Ava-Tex (Cardinal-Health Inc., Dublin, OH, USA)) in our orthopaedic department.

The patient had previously been treated with kyphoplasty of her L5 vertebrae in 2012 and PV at T12 for osteoporotic compression fractures, under local anesthesia, without any complication.

40 minutes after the procedure she started suffering back and leg pain, having repetitive myoclonic jerks lasting 15 seconds of the lower extremities, spasm of the back, dyspnea, and sinus tachycardia. Blood pressure was 132/84 mmHg and temperature 36.2°C. Oxygen saturation (SpO_2_) subsequently began to decrease. The patient was resuscitated according to advanced life support principles and transferred to our intensive care unit to carefully monitor her condition. Arterial blood gas analysis (ABG) showed hypoxemia (PaO_2_ 68 mm Hg) with a metabolic acidosis (pH 7.17, PaCO_2_ 38 mm Hg, HCO_3_ 13.9 mEq/L, BE −14.6 mmol/L, and lactate 8.3 mmol/L). Due to the severe hypoxemia, she was intubated and mechanically ventilated, using fentanyl 200 mcg/propofol 160 mg/atracurium 0.5 mg/kg. Her laboratory findings showed only mild elevation of CK (267 UI/L), with no signs of inflammation (8,800 leukocytes/mm^3^, procalcitonin 0.4 ng/mL, and CRP 3 mg/L). Intravenous (IV) therapy was administered using ampicillin/sulbactam 3 g tds, methylprednisolone bolus 30 mg/kg, clonazepam 5 mg tds, and levetiracetam 1.5 g bd. IV infusion of bicarbonate was required to correct the severe acidosis. The patient was nursed in supine position with the patients head and trunk elevated. After 5 minutes of treatment the spasm regressed but could be elicited by touching the patient. ABG was repeated after 45 minutes, showing a pH of 7.34, PaO_2_ 75 mm Hg, PaCO_2_ 53 mm Hg, HCO_3_ 28.6 mEq/L, BE 2.8 mmol/L, and lactate 4.8 mmol/L. Infusion of propofol 0.075 mg/kg/min, fentanyl 2 mcg/kg/h, methylprednisolone 5.4 mg/kg/h, and crystalloid fluids at 10 mL/kg/h was maintained.

On further investigation computer tomography (CT) of the spine showed epidural cement leakage into the right epidural space at the L2 vertebral level (Figures 1(b) and 1(c)). EEG showed generalized spike wave activity. Head CT scan showed no signs of acute pathology. Considering ongoing hypoxia a CT angiogram of the lungs was performed which showed small bilateral pleural effusion.

Contrast MRI 8 confirmed epidural leakage of PMMA at the L2 level, with enhancement along cauda equina (Figures 1(d) and 1(e)).

The fraction of inspired oxygen (FIO_2_) was maintained at 70% for 7 hours and then gradually reduced to 40% in the subsequent 17 hours. Mean SpO_2_ was 95% in the first 10 hours and PaO_2_ gradually increased. The first attempt to lighten the sedation was made at 10 hours postoperatively but the myoclonic spasms persisted and so deep sedation was continued.

48 hours after the procedure another attempt was made. EEG was repeated and tactile stimulation did not evocate changes on EEG pattern or muscle spasm. Following this the patient was reexamined and found to have no focal neurological signs and was weaned from mechanical ventilation. The patient was then stepped down from the high dependency setting onto the orthopaedic ward with subsequent chest X-ray showing a resolution of the pleural effusions. Laboratory results were all normal. Seven days after surgery the patient was discharged with minimal residual pain (visual analogue scale: 2) and no neurological symptoms.

## 3. Discussion

Vertebral compression fractures occur in 20% of people over the age of 70 years and in 16% of postmenopausal women [5] and are a leading cause of disability and morbidity in the elderly. Cement leakage is a common finding after vertebroplasty, ranging from 11% to 76% of cases [2] in the literature. Cement can leak into epidural, foraminal, intradiscal, intradural, paravertebral, and venous area [6]. Small cement leaks following this procedure are inconsequential. Larger leaks may however produce clinical symptoms such as local or radicular pain, neurologic complications, and pulmonary embolism [7]. Leakage of bone cement accounts for most symptomatic complications following PV [7].

We herein describe a case of ATCS after PV. To our knowledge, this is the first case published in the literature.

We hypothesize a direct MMA monomer toxicity due to leakage into subarachnoid space during the process of polymerization, excluding any damage caused by a thermal injury, as Ava-Tex has a low peak temperature [8]. This syndrome resembles strychnine poisoning. The release of MMA monomers into bloodstream is a potential cause of adverse general reaction, and several MMA esters show a strychnine-like activity [9]. As in the case described by Godoy et al. [10], the effect of the MMA monomers on inhibitory neurons could have led to loss of inhibitory function in the spinal cord determining an impairment of the glycine/GABA mediated inhibitory influence of Renshaw cells on motor neurons.

The symptoms described in our report were identical to those patients who received inadvertent intrathecal injection of ionic contrast media for myelography [4]. Here symptoms started between 1/2 hour and 6 hours after the procedure with initial myotonic and later clonic spasmodic symptoms. These symptoms lasted for 15–30 seconds separated by pain-free intervals. The key feature of this case was that the spasm was elicited by external stimuli, a pathognomonic sign of this syndrome. This prevented the patient from having a second operation for bone cement removal. The patient recovered completely as a result of early intervention. This included mechanical ventilation and neuromuscular paralysis, elevation of head and trunk, aggressive control of seizures, steroids, and prophylactic antibiotics.

ATCS secondary to cement leakage after PV is a very rare phenomenon. This is the first case to our knowledge that has been reported. Prompt identification and management of ATCS is the key to successful treatment.
